# Transport and Metabolism Behavior of Brazilein during Its Entrance into Neural Cells

**DOI:** 10.1371/journal.pone.0108000

**Published:** 2014-10-02

**Authors:** Shuang Zhao, Xin-Pei Wang, Jing-Fei Jiang, Yu-Shuang Chai, Yu Tian, Tian-Shi Feng, Yi Ding, Jing Huang, Fan Lei, Dong-Ming Xing, Li-Jun Du

**Affiliations:** 1 MOE Key Laboratory of Protein Sciences, Laboratory of Molecular Pharmacology and Pharmaceutical Sciences, School of Life Sciences and School of Medicine, Tsinghua University, Beijing, China; 2 Drug Discovery Facility, School of Life Sciences, Tsinghua University, Beijing, China; 3 Department of Chemistry, Virginia Polytechnic Institute and State University, Blacksburg, Virginia, United States of America; University of Medicine & Dentistry of NJ - New Jersey Medical School, United States of America

## Abstract

Brazilein, a natural small molecule, shows a variety of pharmacological activities, especially on nervous system and immune system. As a potential multifunctional drug, we studied the distribution and the transport behavior and metabolic behavior of brazilein in vivo and in vitro. Brazilein was found to be able to distribute in the mouse brain and transport into neural cells. A metabolite was found in the brain and in the cells. Positive and negative mode-MS/MS and Q-TOF were used to identify the metabolite. MS/MS fragmentation mechanisms showed the methylation occurred at the10-hydroxyl of brazilein (10-O-methylbrazilein). Further, catechol-O- methyltransferase (COMT) was confirmed as a crucial enzyme correlated with the methylated metabolite generation by molecular docking and pharmacological experiment.

## Introduction

**Figure 1:**
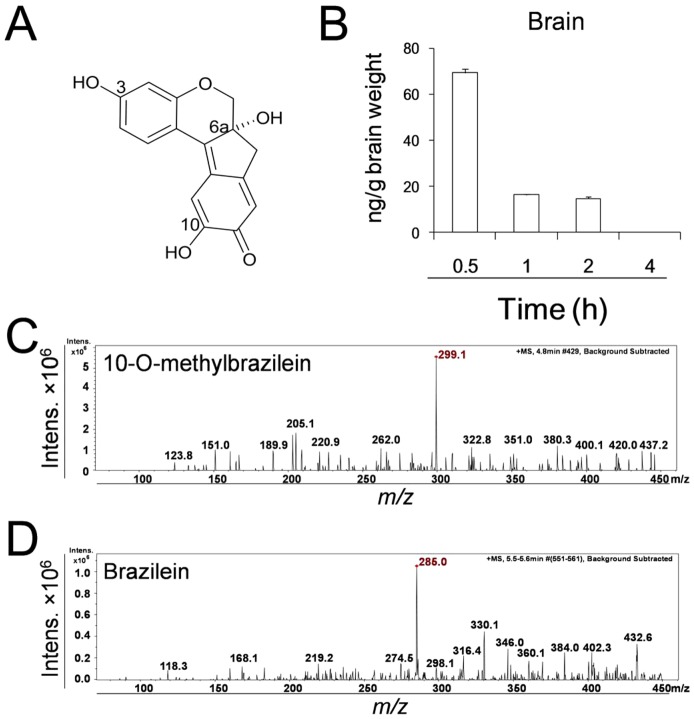
Distribution of brazilein in mice brain and mass spectrum of brazilein and its motabolite. (A) Chemical structure of brazilein. (B) Time course of brazilein contents in mice brain. (C) Mass spectrum of brazilein (*m/z* 285) in mice brains. (D) Mass spectrum of the motabolite (*m/z* 299) in mice brains. The dose of brazilein was 5 mg/kg using introvenouse injection. Data were presented as mean ±S.D. from six independent mice (n = 6).

Brazilein (6a,7-dihydro-3,6a,10-trihydroxy-benz[b]indeno[1,2-d]pyran-9(6H)-one, Fig. 1A) is a natural small molecule isolated from dried heartwood of *Caesalpinia sappan* L [1]–[3]. It has been reported that brazilein exhibits multi-pharmacological activities, such as cardioactive effect [4], immunosuppression [5], [6], protection of central and peripheral nerves system [7]–[9], smooth muscle contraction promotion [10], melanin synthesis suppression [11], anti-oxidant [12], [13] and anti-influenza viral activities in vitro [14]. The neural protection effect of brazilein after ischemia/reperfusion injury was studied systematically. It has been reported that this function is correlative with the inflammation suppression effect of brazilein. In the previous research, brazilein was observed that it can inhibit pro-inflammatory cytokine activation, suppress NO production, and inhibit iNOS and cytokine expression [8], [9]. Brazilein was also reported to work on 5-HT receptors as an antagonist [15]. Recent studies of brazilein mostly focus on its anticancer activity [16]–[19]. All of these remind us that brazilein is a potentially valuable drug to be developed.

The pharmacokinetic study in plasma showed that the distribution of *t*_1/2α_ was 16.89 min, and clearance of *t*_1/2β_ was 280.07 min after intravenous administration of brazilein [20]. Based on previous results, it is necessary to study brazilein's cerebral distribution and transportation through neural cells, in order to well understand the mechanism of brazilein on cerebral protection.

In this research, mice and PC12 cells were used as the models in vivo and in vitro, respectively. HPLC system and confocal microscopic photographs were used to detect the distribution and transportation of brazilein. The distribution of brazilein in the mouse brain was observed and the transport behavior of brazilein through neural cells in vitro was described. A new single substituted metabolite in the mouse brain and in the cell was identified with LC-MS/MS. The related enzyme of this metabolite was confirmed by molecular docking and pharmacological experiment.

## Materials and Methods

### Animals

Male ICR mice (25–28 g), purchased from Vital River Laboratories (Beijing, China), were kept in the Animal Center of Tsinghua University. Mice were maintained in an environmentally controlled breeding room (temperature 25°C, relative humidity 45–55%, 12 h light/dark cycle). They were fed with standard food pellets and tap water ad libitum. The laboratory animal facility has been accredited by AAALAC (Association for Assessment and Accreditation of Laboratory Animal Care International) and the IACUC (Institutional Animal Care and Use Committee) of Tsinghua University approved all animal protocols used in this study (Approval ID: 2013-DuLJ001).

### Cells and reagents

PC12 cells were commercially obtained from the Institute of Basic Medical Sciences, Chinese Academy of Medical Sciences. Methanol and acetonitrile (chromatographic grade), were purchased from Fisher (Germany). All other reagents with analytical grades were purchased from Beijing Chemical Plant.

### Experiment procedures

#### Experiment 1: Brazilein distribution in mice brain

Experimental mice were intravenously administered with brazilein, which was solved in DMSO and then diluted with sterile normal saline solution at a dose of 10 mg/kg (the final concentration of DMSO was controlled less than 1/1000 of concentration). Normal saline with DMSO (same as brazilein groups) served as vehicle control. Then blood and brain samples were taken at 30, 60,120, and 240 min after brazilein administration. The blood samples mixed with heparin were then centrifuged at 4000 rpm for 10 min to get plasma. The brain samples were grinded with saline (pH = 8) to reach homogenate. Both plasma and brain homogenate were extracted three times with three times the amount of ethyl acetate (ethyl acetate: sample  = 3: 1). Then combine and evaporate the supernatant at room temperature. The resulting residue was re-dissolved in 100 µL methanol to determine brazilein and its metabolite with HPLC/MS/MS.

#### Experiment 2: Drug administration and sample preparing

Brazilein was dissolved in DMSO and diluted with serum-free medium in experiments (DMSO was controlled less than 1/1000 of concentration). The cells used in experiments were cultured to reach a density of 70% and then incubated with serum-free culture medium containing different concentrations of brazilein. After the incubation finished, the drug-containing medium was evaporated, and the cells were washed three times with cold PBS (4°C). After 1.2 mL methanol added, the cells were collected into EP tubes by cell scraper and broken with an ultrasonic cell disruptor. The lysates were centrifuged at 10000 rpm for 5 min and the supernatant was collected. The precipitates were extracted with methanol containing 10% triethylamine three times. The liquid phase was combined with the supernatant and evaporated with centrifugal concentrator system (Labconco, U.S.) at room temperature. The residue was re-dissolved in 100 µL of methanol to determine the content of brazilein with HPLC. The precipitates in each tube were re-dispersed in PBS to quantify the protein content by the method of Bradford using bovine serum albumin (BSA) as a standard [21].

#### Brazilein transport behavior analysis

Different conditions were applied in order to study brazilein transportation behavior. The cells were administrated with brazilein of different administration doses (0 to 15 µg/mL), at different temperatures (28, 37 and 40°C), with metabolism inhibitor (KCN, Sigma-Aldrich, U.S.) and with catechol-O-methyltransferase inhibitor (entacapone, Selluck Chemicals, U.S.), respectively. Intracellular and extracellular concentrations of brazilein and its metabolite were detected. The intracellular concentration was depicted by the ratios of values determined using HPLC to the protein content determined via Bradford method [21].

### Cell culture

PC12 cells were cultured in RPMI1640 at 37°C. The medium, without phenol red, included 10% fetal bovine serum and 5% horse serum.

### MTT assay for cytotoxicity

The cytotoxicity of brazilein in PC12 cells was determined using an MTT assay. The MTT assay was operated according to the reference 8.

### HPLC/MS System and Conditions

The HPLC system (Waters, U.S.) consisted of a 515 HPLC pump, a 996 Photodiode Array Detector, a Rheodyne 7725i manual injector, and the Empower2 Working Station. Separation was carried out with an XTerra RP_18_ column (5 µm; 3.9×150 mm, Waters). The mobile phase was acetonitrile - water (containing 0.1% formic acid, 10 mM ammonium acetate; 30: 70 v/v). The flow rate was 0.4 mL/min. Detection was performed under a constant temperature (25°C) at the wavelength of 445 nm.

The LC-MS/MS detection was performed with Agilent 1200/6340 linear ion-trap LC/MS system. The HPLC condition was the same with that described previously. MS/MS analysis was operated in both positive ion mode and negative ion mode. The parent ions m/z and fragmentation patterns were analyzed to determine compounds.

Q-TOF detection was carried out with Waters Q-TOF LC/MS, Xevo G2 system. The HPLC condition was described as above and MS detection was conducted in positive ion mode.

### Confocal microscopy

PC12 cells were used in experiments when the density reached 70%. Brazilein - treated (5 µg/mL) cells and the control group, no-brazilein-treated cells, were then fixed with 4% paraformaldehyde. Propidium iodide (PI) was used to stain the nucleus of fixed cells. Images were taken with Zeiss LSM 710 Confocal Microscope (Carl Zeiss, Germany) and analyzed using Zen 2009 Light Edition Software. Brazilein and PI were excited at 490 nm and 536 nm, respectively.

### Molecular docking

Molecular docking was performed with Autodock 4.2 and presented by PyMOL Molecular Graphics System. The 3D structures of protein COMT with cofactors and brazilein were downloaded from RCSB Protein Data Bank (PDB code: 1H1D) and Pubchem-NCBI, respectively.

### Statistical analysis

Data are expressed as mean ±S.D. Data were statistically analyzed using one-way analysis of variance (ANOVA) with F value determination. The F test was carried out using Excel software for Office 2007 (Microsoft, U.S.). The student's *t*-test between two groups was performed after the F test. *P* values below 0.05 were considered statistically significant.

## Results and Discussion

### Distribution of brazilein

30 min after intravenous administration, brazilein can be found in the mouse brain. And then, it decreased quickly. 4 h after the administration, brazilein was unable to be detected in the mouse brain (Fig. 1B). By using LC-MS, the fragments of brazilein (*m/z* 285) in the mouse brain were determined (Fig. 1C). Meanwhile, a small amount of metabolite (*m/z* 299) was detected in the mouse brain using LC-MS (Fig. 1D).

### Brazilein can be detected by HPLC-UV

**Figure 2:**
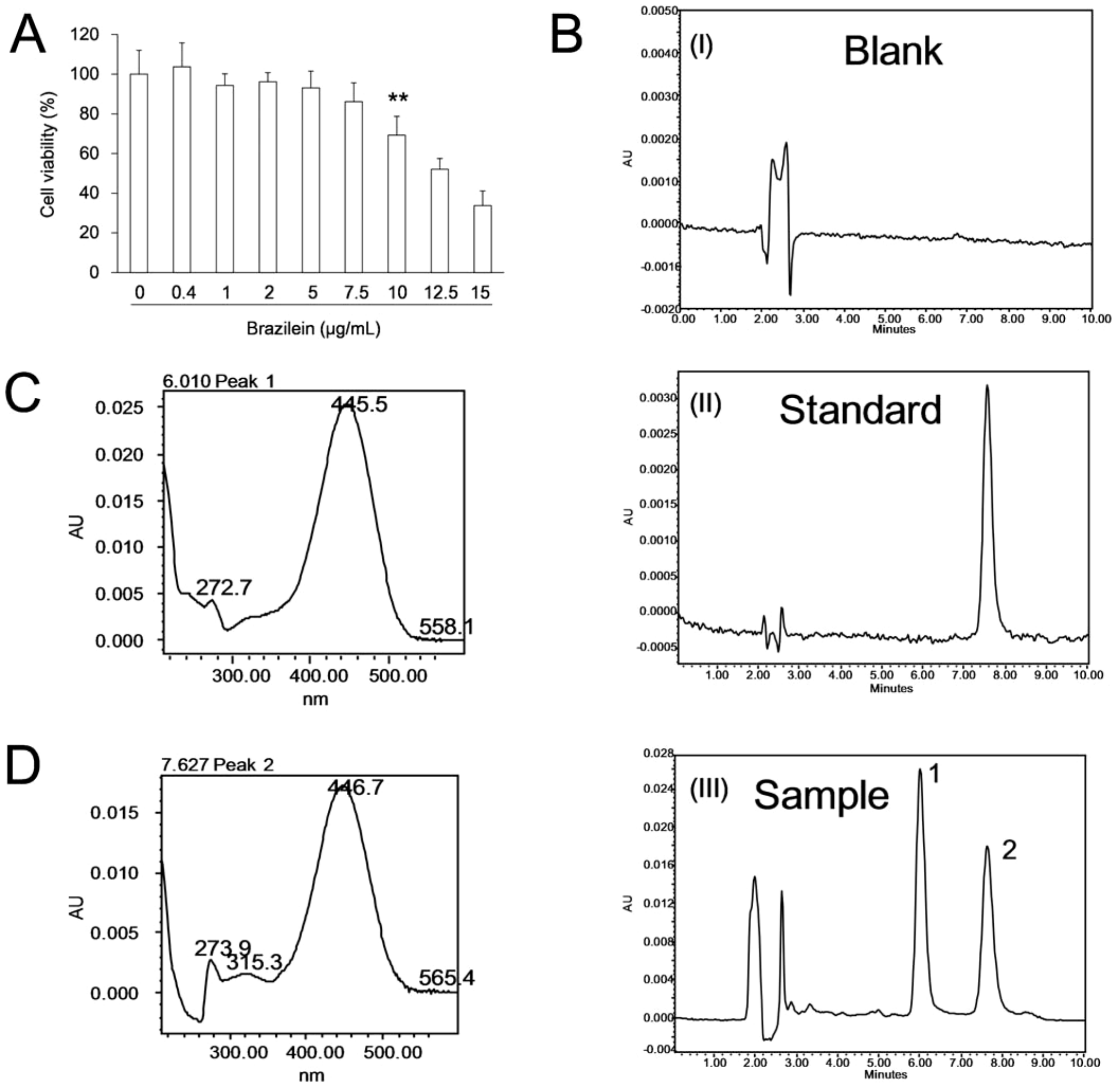
Cytotoxicity assay and HPLC detection of brazilein. (A) The chemical structure formula of brazilein. (B) Cytotoxicity of brazilein in the MTT assay. In the assay, the group with 0 µg/ml brazilein was considered as the control. Data were presented as mean ±S.D. from six independent experiments (n = 6). ** *p*<0.01 *v.s.* the control. (B) The HPLC chromatograms of brazilein: B (I) is the chromatograms of cell lysate as the blank control; B (II) represents the brazilein standard; B (III) represents the cells administrated with 5 µg/mL brazilein for 4 h. Peak 2 in B (III) shares the same retention time with brazilein standard while peak 1 is ahead of the standard. (C) UV spectrogram of peak 1. (D) UV spectrogram of peak 2 (brazilein standard).

The MTT assay showed that 10 µg/mL brazilein produced significant cytotoxicity to PC12 cells in 24 h (Fig. 2A). Therefore, the safe dose of 5 µg/mL was used in experiments. Under the HPLC conditions described in methods, the chromatographs of blank cells without brazilein, brazilein standards and the test sample in which the cells incubated with 5 µg/mL brazilein for 4 h, were shown in Fig. 2B. The chromatograph of the test sample indicated that an unknown compound was produced after the administration of brazilein (Fig. 2B(III) peak1). The UV-VIS absorption spectrum of this unknown compound was similar to that of brazilein (Figs. 2C–D), which implied that this compound might be a structural analogue of brazilein (Fig. 2B(III) peak2). It is named brazilein-X temporarily. The retention time of brazilein-X and brazilein were 6.01 min and 7.63 min, respectively. This indicates that the brazilein-X is more polarity than brazilein. Besides these two peaks, there is no other peak on HPLC graph (up to 60 min).

The calibration curve was plot linear of HPLC peak areas over the concentration range of 0.1, 0.2, 0.5, 1, 2 and 5 µg/mL of brazilein. The correlation coefficient (*r* ^2^) was 0.9994, indicating a good linear relationship between peak areas and brazilein concentrations. The relationship was quantified by the equation: *y* = 4.88×10^6^ *x*–3845.23, where *x* represented the concentration of brazilein and *y* represented the peak areas. The intra-day and inter-day precision were evaluated using three different concentrations (Table 1 and Table 2). Maximal CV value was 2.59% for intra-day and 5.29% for inter-day precision, indicating HPLC is quite a precise detection method for brazilein. The recovery of brazilein was determined by comparing the data obtained by standard with the same concentration brazilein after the whole extraction procedure described in methods. The average recovery was 46.77%.

**Table 1:** Validation of the intra- and inter-day precision of brazilein.

Spiked concentration (µg/ml)	Measured concentration (µg/ml)^a^	Accuracy (%)	CV (%)
*intra-day*			
0.2	0.189±0.005	94.58	2.59
1	0.953±0.018	95.25	1.82
5	4.877±0.067	97.53	1.33
*inter-day*			
0.2	0.184±0.010	91.81	5.29
1	0.968±0.034	96.79	3.42
5	4.861±0.130	97.21	2.60

a Each value represents the mean ± S.D. (n = 3).

**Table 2:** Recovery of brazilein.

Spiked concentration (µg/ml)	Measured concentration (µg/ml)^a^	Recovery (%)	CV (%)
0.2	0.097±0.007	48.53	6.95
1	0.462±0.017	46.22	3.73
5	2.278±0.124	45.55	5.44

a Each value represents the mean ±S.D. (n = 3).

### Brazilein in PC12 cells

**Figure 3:**
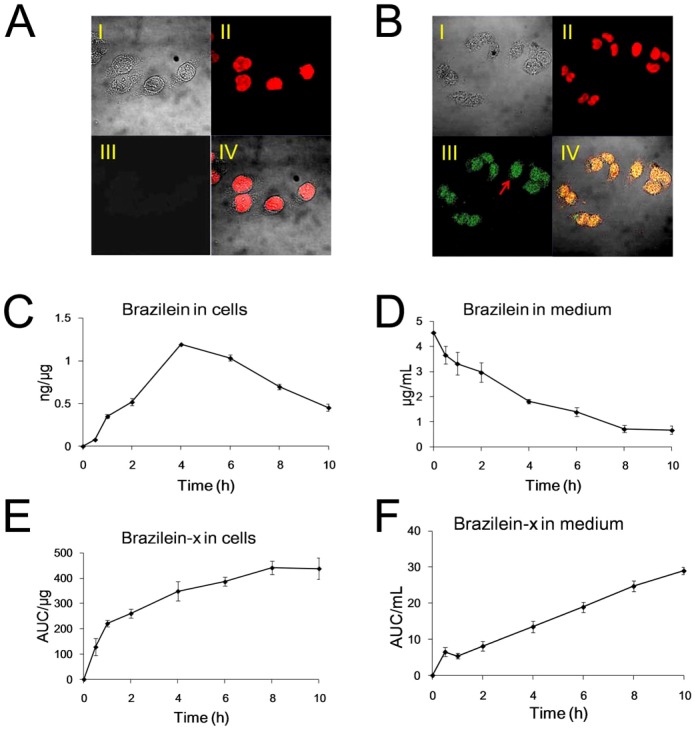
Entrance of brazilein into cells. (A) Confocal microscopy image of cells without brazilein. (B) Image of cells administrated with 5 µg/mL brazilein for 4 h. A (I) and B (I) represent PC12 cells in bright field; A (II) and B(II) represent the nucleus stained by PI; B (III) represents the fluorescence of brazilein; (IV) is the merged image of (I), (II) and (III) in A and B, respectively. (C–D) Concentration-time curve of brazilein in cells and in medium. (E–F) Concentration-time curve of brazilein-X in cells and in medium. Brazilein was added to the culture with concentration of 5 µg/mL. Data were presented as mean ±S.D. from three independent experiments (n = 3).

We used confocal assay to detect the entrance of brazilein into PC12 cells. Brazilein emits green fluorescence at an excitation wavelength of 449 nm. In the experiments, the green fluorescence can be observed after 1 h of brazilein (5 µg/mL) into the medium, and it's getting more obviously at 4 h. (Figs. 3A–B).

The concentration of intracellular and extracellular brazilein was quantified by HPLC. The concentration versus time curve of brazilein in cells showed that the intracellular brazilein reached the maximum at 4 h, and then gradually decreased (Fig. 3C). Meanwhile, the concentration of extracellular brazilein decreased (Fig. 3D). The negative control (medium containing brazilein but without cells) exhibited that concentration of brazilein did not change over time and no new metabolites were produced. This suggested that brazilein indeed entered the cell, therefore decreased the extracellular concentration and increased the intracellular concentration. While brazilein was detected in the cells, a metabolite (named brazilein-X temporarily) can be found. Along the time, the intracellular metabolite increased, as well as the metabolite in the medium increased (Figs. 3E–F). It is suggested that the metabolite was generated in the cells and discharged into the medium.

### Brazilein enters cells by passive transportation

**Figure 4:**
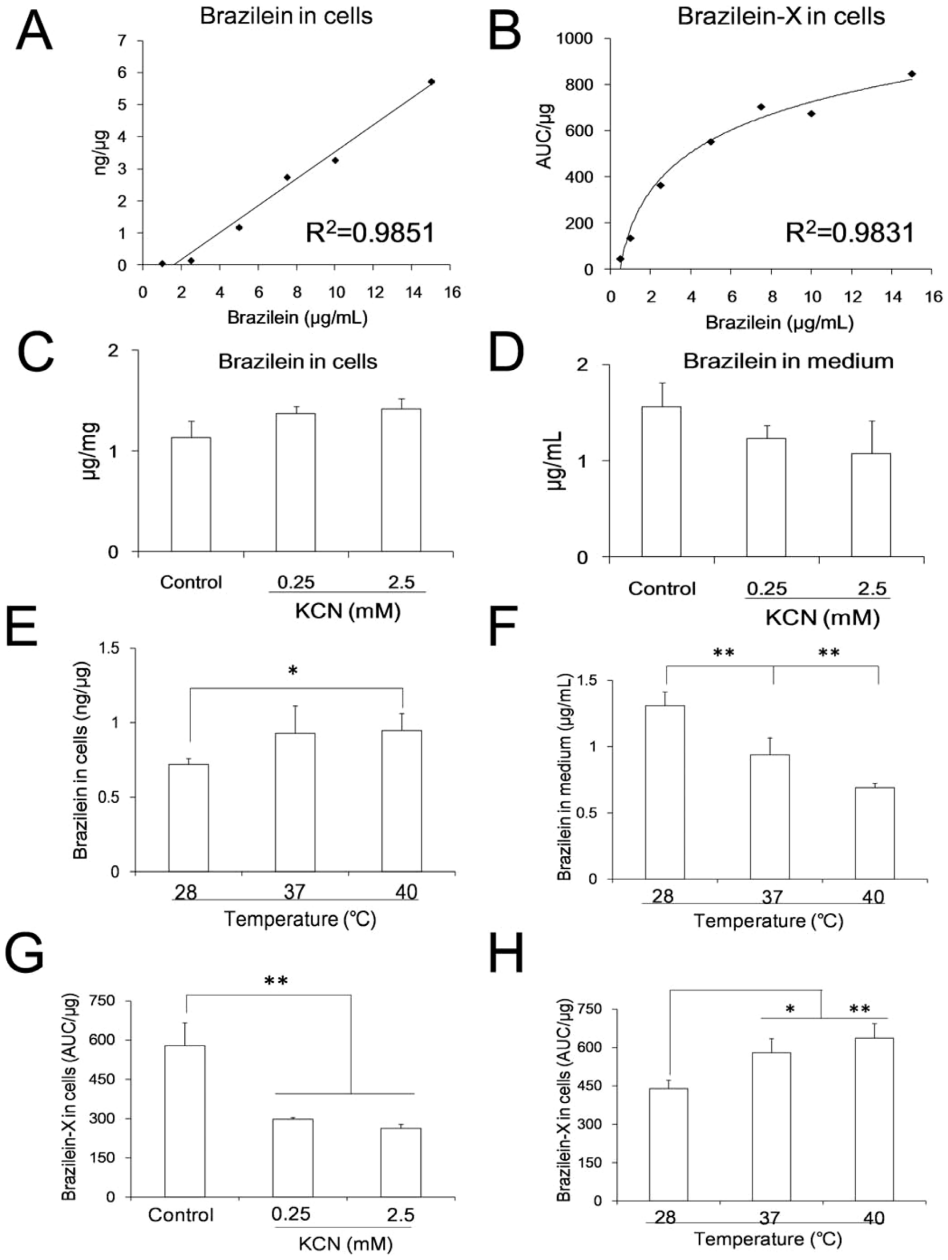
Effects of administrated dose, inhibitor of cytochrome oxidase and temperature on transportation and metabolism of brazilein. (A) – (B) Concentrations of brazilein and brazilein-X in cells when cells were administrated with different doses of brazilein for 4 h. Linear fitting and logarithm fitting are used to analyze data in (A) and (B). (C) – (D) Effect of cytochrome oxidase inhibitor (KCN) on concentration of brazilein in cells and in medium. (E) – (F) Effect of temperature on concentration of brazilein in cells (E) and in medium (F). (G) Effect of cytochrome oxidase inhibitor (KCN) on concentration of brazilein-X in cells. (H) Effect of temperature on concentration of brazilein-X in cells. In Figures (C) – (H), cells were administrated with 5 µg/mL brazilein for 4 h. Data were presented as mean ±S.D. from three independent experiments (n = 3). * *p*<0.05, ** *p*<0.01 *v.s.* controls.

In our prior MTT assay experiment, 20 µg/mL brazilein would produce significant cytotoxicity to PC12 cells in 4 h, and 15 µg/mL brazilein was the safe dosage without any cytotoxicity to the cells (Fig. S1 in File S1). Therefore, we choose 15 µg/mL brazilein as a safety dosage in the 4 hour-experiment. In the experiment, the intracellular concentration of brazilein increased in a concentration-dependent manner (brazilein range of 0 to 15 µg/mL), the correlation coefficient was 0.9851 (Fig. 4A). This indicated that brazilein did not appear transport saturation phenomenon within the dose range, while the saturation might occur in the process of active transportation or endocytosis because of the restrictions of the transporter numbers.

When KCN was introduced to cells as an energy generation inhibitor, it was showed that either low concentration (0.25 mM) or high concentration (2.5 mM) of KCN did not significantly change the content of brazilein in cells or in medium (Figs. 4C–D). This suggested that the entrance of brazilein is an energy-independent process, implying that the transportation of brazilein was not an active transport or endocytosis process but a passive transportation. Under the high temperature (40°C), the intracellular brazilein was found to significantly increase, while extracellular brazilein decreased (Figs. 4E–F). This indicated that brazilein transport process was temperature-dependent.

When the administrated brazilein increased, the generation of brazilein-X increased before reaching at a plateau region (Fig. 4B). The correlation coefficient of the logarithmic fitting is 0.9831. KCN also significantly inhibited brazilein-X generation (Fig. 4G). In addition, under 37°C and 40°C, the content of intracellular brazilein-X has significantly exceeded that under 28°C (Fig. 4H). These results indicated that brazilein-X was generated in dose-dependent, energy-dependent and temperature-dependent manners.

### Identification of the metabolite with Ion trap MS and Q-TOF MS under positive and negative mode

**Figure 5:**
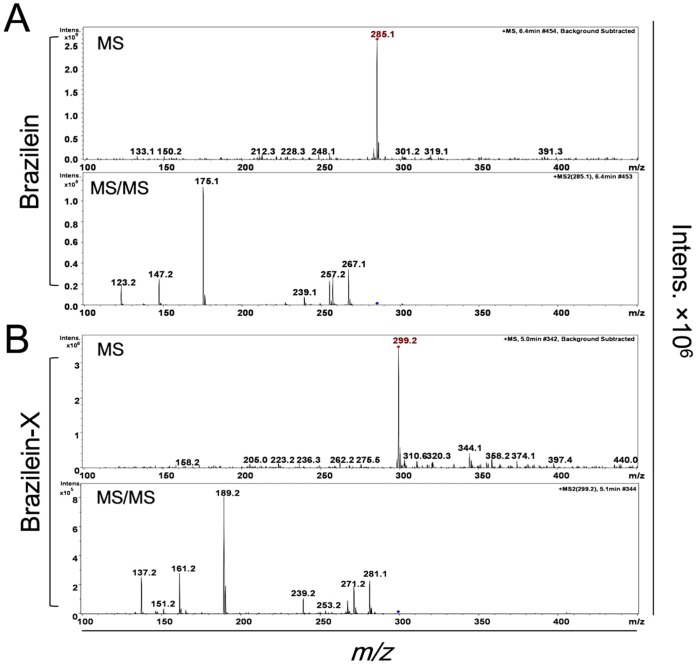
Mass spectrum (MS, MS/MS) of brazilein and brazilein-X in positive ion mode. (A) Mass spectrum of brazilein (*m/z* 285). (B) Mass spectrum of brazilein-X (*m/z* 299).

LC/MS analysis showed that the molecular weight of the metabolite brazilein-X was 299, which was 14 more than the molecular weight of brazilein (Fig. 5). The MS/MS results showed that brazilein-X shared the same fragmentation pattern with brazilein in positive ion mode, and the *m/z* of each fragment of brazilein-X was 14 more than the corresponding fragment of brazilein. In the negative mode of MS/MS, the quasi-molecular ion peaks of brazilein-X lost a fragment with molecular weight of 15. It can be speculated that this new metabolite was a methylation of brazilein (methyl-brazilein). We used high resolution MS (Waters Q-TOF LC/MS, XevoG2) to identify the methylation of brazilein. The mass spectra were matched with our previous results (Figs. S2 and S3 in File S1). We used the Elemental Composition Analysis Function of the mass spectrometry to characterize the metabolite. This elemental composition analysis is based on isotope ratio. It showed that compared with brazilein, the metabolite had one more carbon and two more hydrogen, which supported our inference of methylation of brazilein (Tables. S1 & S2).

**Figure 6:**
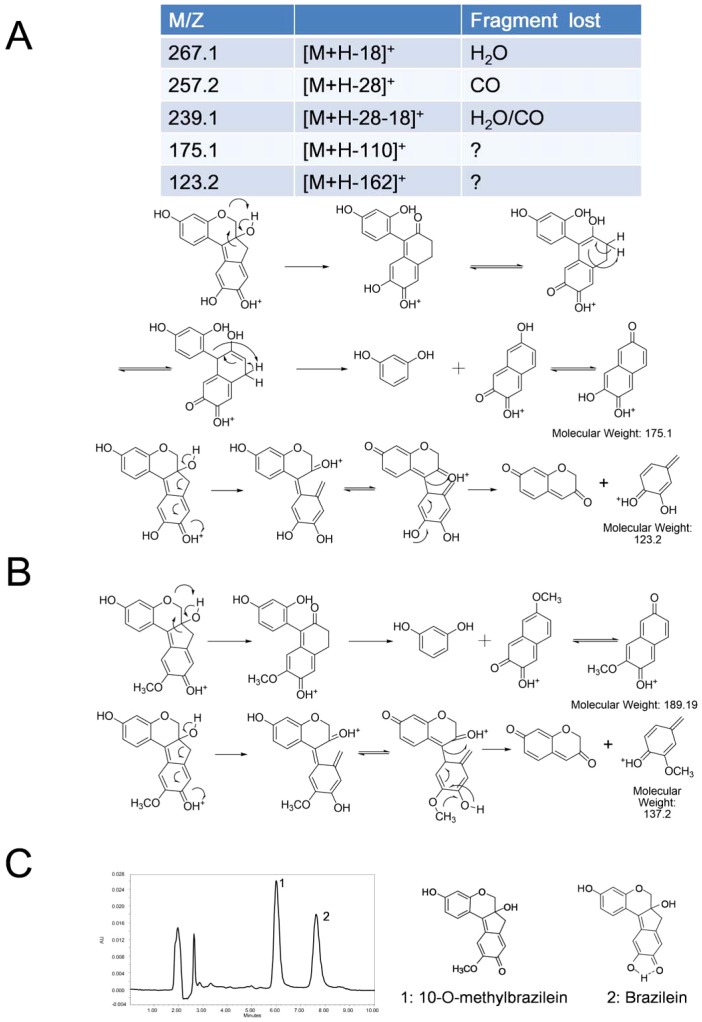
MS analysis of brazilein and its metabolitebrazilein-X (10-O-methylbrazilein). (A) Positive ion ESI mass spectra and fragmentation of brazilein. (B) Fragmentation of methyl-brazilein. (C) HPLC chromatogram of 10-O-methylbrazilein and brazilein.

There are three hydroxyl groups that are the potential methylation sites in brazilein. To determine the location of methylation, we proposed the fragmentation mechanisms via positive ion mode of MS/MS (Fig. 6A) in the work by Hulme *et al.* in 2005 [22]. They rationalized the fragmentation mechanism of brazilein and some analogues, but without the fragmentation of methyl-brazilein under positive ESI. The *m/z* of 189 in methyl-brazilein is proposed to follow the same fragmentation mechanism of *m/z* 175 of brazilein. The *m/z* of 137 in methyl-brazilein is proposed to follow the same fragmentation mechanism of *m/z* 123 of brazilein (Fig. 6B). The methylation site and the chemical structure were therefore determined and the new metabolite might be determined to be 10-O-methylbrazilein (Fig. 6C).

The phenomenon that the peak of 10-O-methylbrazilein appeared earlier than the peak of brazilein in the HPLC chromatogram could support the inference of 10-O-methylbrazilein (Fig. 6C). As a general rule, methylation of a hydroxyl group decreases the molecular polarity. Then the retention time in the RP-HPLC will increase, which is contradicted with the observed phenomenon in our research. These could be explained that the intra-molecular hydrogen bond formed between 10-hydroxyl group and the adjacent carbonyl group in brazilein, causing the molecular polarity reduced. Methylation process was able to break this hydrogen bonding, increasing the polarity and shortening the retention time.

In previous chemical research, some of its analogues have been isolated from *Caesalpinia sappan* L, including brazilin (the hydrogenation form of brazilein), 3′-O-methylbrazilin and neoprotosappanin [23]. In 2009, Yen, C. T. *et al.* reported the total synthesis of brazilein and its derivatives of replacing the hydroxyl groups in brazilein and brazilin [24]. Disubstituted and trisubstituted derivatives, such as trimethyl brazilin [25], have been synthesized [26]. But region selective single substituted compounds have not been reported yet. Previous structure-activity research of brazilein in our laboratory showed that it was difficult to selective replace a single hydroxyl group by chemical methods because of the similarity between 3-hydroxyl and 10-hydroxyl in brazilein.

10-O-methylbrazilein, as a single-substituted derivative, was not reported in the previous studies. Research groups rarely reported single-substituted derivatives of brazilein by chemical reaction [27], [28]. Thus, the chemical properties and biological activities of this new analogue were yet unknown. The issue merits further study.

### COMT contributes to the methylation of brazilein

**Figure 7:**
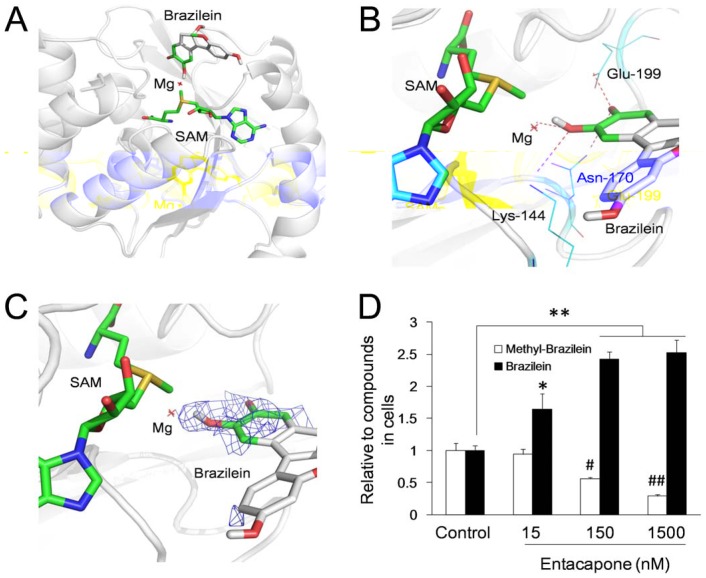
Methylation of brazilein by catechol-O-methyltransferase (COMT). (A) Molecular Docking model of brazilein and COMT with cofactors S-adenosyl methionine (SAM, served as methyl donor) and Mg^2+^ (PDB code: 1H1D). The white cartoon represents COMT. Red dot represents Mg^2+^. SAM and brazilein are showed as stick structure (colored by atom type: greencarbon, redoxygen, bluenitrogen, yellowsulfur). (B) Hydrogen bonds between brazilein and COMT with Mg^2+^. Residues linking to brazilein by hydrogen bonds are labeled and showed of blue lines. Red line of dashes represents the hydrogen bonds. (C) The electron density map of COMT around brazilein. Molecular docking was performed using Auto-Dock Vina.Figures were drawn using PyMOL Molecular Graphics System. (E) Effect of COMT inhibitor (entacapone) on transformation of brazilein to methyl-brazilein (10-O-methylbrazilein). Cells were administrated with 5 µg/mL brazilein for 4 h. Data were presented as mean ±S.D. from three independent experiments (n = 3). * *p*<0.05, ** *p*<0.01 *v.s.* controls. # *p*<0.05, ## *p*<0.01 *v.s.* controls.

Computer-based molecular docking was conducted to further demonstrate the inferences above and to understand the enzymatic reaction of brazilein methylation. The results showed that brazilein was able to combine with the active sites of catechol-O-methyltransferase (COMT) and the S-adenosyl methionine (SAM), which is a common co-substrate involved in methyl group transfers and served as methyl donor (Fig. 7A, PDB code: 1H1D). The hydrogen bonds between brazilein and COMT predicted by docking were showed in Fig. 7B. It is showed that Lys144 and Mg^2+^ ion linked with the 10-hydroxyl which was methylated in brazilein, and the carbonyl group adjacent to the 10-hydroxyl had hydrogen bonds with carboxylate of Glu199 and amidogen of Asn170. Brazilein is able to well fit into the electron density map of COMT (Fig. 7C). The detailed analysis with docking predicted that brazilein could combine with the COMT similarly with the reported substrates and inhibitors of COMT [29], [30].

In the experiments, different concentrations (15, 150, 1500 nM) of entacapone, an inhibitor of COMT, were administrated to PC12 cells with 5 µg/mL brazilein, in order to confirm the relationship between COMT and brazilein metabolite. Results showed that entacapone significantly inhibited the transition of brazilein to metabolite and rendered in a dose-dependent manner (Fig. 7D). These results supported the computer's prediction, indicating that COMT could methylate brazilein into 10-O-methylbrazilein.

Common substrates of catechol-O-methyltransferase are catechol derivatives, including endogenous molecules such as dopamine, epinephrine and norepinephrine [31] and varied exogenous compounds like 3,5-dinitrocatechol and catechol containing adenine replacement [32]. Tolcapone and entacapone which was used in this research have been used as inhibitors of COMT in the therapy of Parkinson's disease [33]. Previous researchers have extensively studied the mechanism of COMT methylation process from structural perspective [30] and functional perspective [34]. Docking model in this research displayed the combination between brazilein and COMT complex with SAM and Mg^2+^ (PDB code: 1H1D), which was corresponding to the proposed catalytic mechanism [27], [35]. From molecular level, brazilein bond to the enzyme active site which is near the surface of the enzyme and was close to the methyl donator SAM and cofactor Mg^2+^. From view of bond level, Lys144, in which NH_2_ acted as the catalytic core to deprotonate hydroxyl in catechol, displayed a connection to the 10-hydroxyl of brazilein through hydrogen bonding. Mg^2+^, Asn170 and Glu199 also had hydrogen bonds with hydroxyl and carbonyl groups for “anchoring” effect.

Though brazilein does not have catechol structure, the computer-based molecular docking and the pharmacological experiment implied that brazilein was a substrate of COMT. This may ascribe to the carbonyl group adjacent to the 10-hydroxyl, which makes the similar spatial structure and bond connection as catechol. It suggested that other compounds with similar structure might also be the substrates of COMT. Because the known COMT competitive inhibitors are almost catechol structure, this discovery may provide a new thought to search and design COMT inhibitors, especially served as drugs for Parkinson's disease. However, this kind of inhibitors may still have similar side effect with existed Parkinson's disease drugs, such as constipation. Tolcapone and entacapone also have this side effect. In addition, biosynthesis with COMT is probably a new method to synthesize the regioselective single-methylated brazilein and may extend to other similar compounds. All these assumptions are remained to be studied.

## Conclusions

Taken together, brazilein is able to distribute in the mouse brain and enter PC12 cells via a passive transportation. The transportation of brazilein was a dose-dependent, non-saturated, energy-independent and temperature-dependent process. During this process, brazilein could be transformed into 10-O-methylbrazilein in the brain and neural cells. COMT contributes to the transformation. These results are of benefit to understand the neural protect effects and the metabolism of brazilein.

## Supporting Information

File S1 **Figure S1,** MTT assay of brazilein for 4 hours. In the assay, the group with 0 µg/mL brazilein was considered as the control. Data were presented as mean ±S.D. from six independent experiments (n = 6). ** *p*<0.01 *v.s.* the control. **Figure S2,** Mass spectrum of brazilein and the metabolite in high resolution MS (Waters Q-TOF LC/MS, XevoG2). **Figure S3,** MS/MS of brazilein and the metabolite in high resolution MS (Waters Q-TOF LC/MS, XevoG2).
(DOCX)

Table S1 **Elemental composition report of brazilein.**
(DOCX)

Table S2 **Elemental composition report of metabolite.**
(DOCX)
