# Supplementary material for: Transport and Metabolism Behavior of Brazilein during Its Entrance into Neural Cells
Source: PLoS One. 2014 Oct 2;9(10):e108000. doi: 10.1371/journal.pone.0108000 (PMC4183444; doi:10.1371/journal.pone.0108000)
Supplement: File S1 — Figure S1, MTT assay of brazilein for 4 hours. In the assay, the group with 0 µg/mL brazilein was considered as the control. Data were presented as mean ±S.D. from six independent experiments (n = 6). ** p<0.01 v.s. the control. Figure S2, Mass spectrum of brazilein and the metabolite in high resolution MS (Waters Q-TOF LC/MS, XevoG2). Figure S3, MS/MS of brazilein and the metabolite in high resolution MS (Waters Q-TOF LC/MS, XevoG2). (DOCX) [file pone.0108000.s001.docx]

Figure S1. MTT assay of brazilein for 4 hours. In the assay, the group with 0 μg/mL brazilein was considered as the control. Data were presented as mean ± S.D. from six independent experiments (n=6). ** *p* < 0.01 *v.s.* the control.

Figure S2. Mass spectrum of brazilein and the metabolite in high resolution MS (Waters Q-TOF LC/MS, XevoG2).

Figure S3. MS/MS of brazilein and the metabolite in high resolution MS (Waters Q-TOF LC/MS, XevoG2).
