# Supplementary material for: Transport and Metabolism Behavior of Brazilein during Its Entrance into Neural Cells
Source: PLoS One. 2014 Oct 2;9(10):e108000. doi: 10.1371/journal.pone.0108000 (PMC4183444; doi:10.1371/journal.pone.0108000)
Supplement: Table S1 — Elemental composition report of brazilein. (DOCX) [file pone.0108000.s002.docx]

Table S1. Elemental composition report of brazilein

|  |  | Elemental Composition Report | | | | |  |  |
| --- | --- | --- | --- | --- | --- | --- | --- | --- |
|  |  |  | Single Mass Analysis | | |  |  |  |
| Tolerance=5.0PPM/DBE:min=-3.0, max=50.0 | | | | | |  |  |  |
| Element prediction: Off | | | |  |  |  |  |  |
| Number of isotope peaks used for i-FIT =3 | | | | |  |  |  |  |
| Element Used: | |  |  |  |  |  |  |  |
| C:0-500 H:0-1000 N:0-200 O:0-200 Na:0-1 P:0-5 S:0-2 | | | | | | |  |  |
| Mass | Calc.Mass | mDa | PPM | DBE | i-FIT | Norm | Conf(%) | Formula |
| 285.076 | 285.0763 | -0.1 | -0.4 | 10.5 | 862.9 | 0.052 | 94.93 | C16H13O5 |
